# Supplementary figures and images for: A gene co-expression network-based analysis of multiple brain tissues reveals novel genes and molecular pathways underlying major depression
Source: PLoS Genet. 2019 Jul 15;15(7):e1008245. doi: 10.1371/journal.pgen.1008245 (PMC6658115; doi:10.1371/journal.pgen.1008245)

A

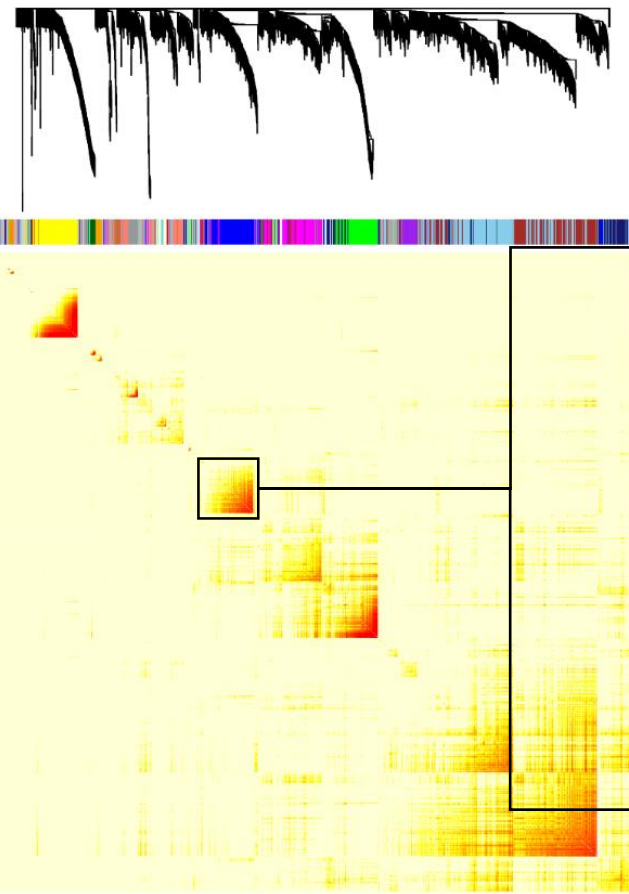

B

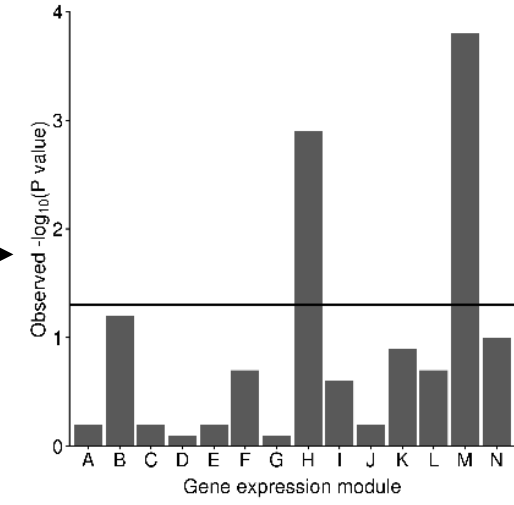

C

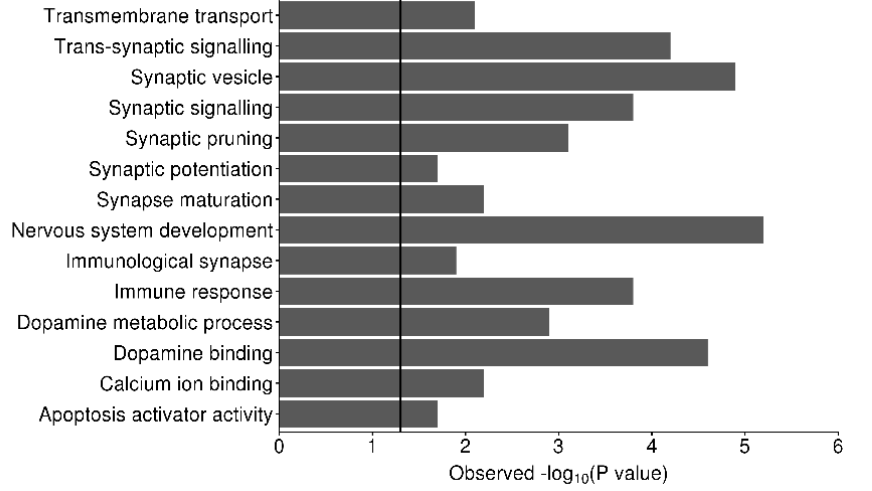

D

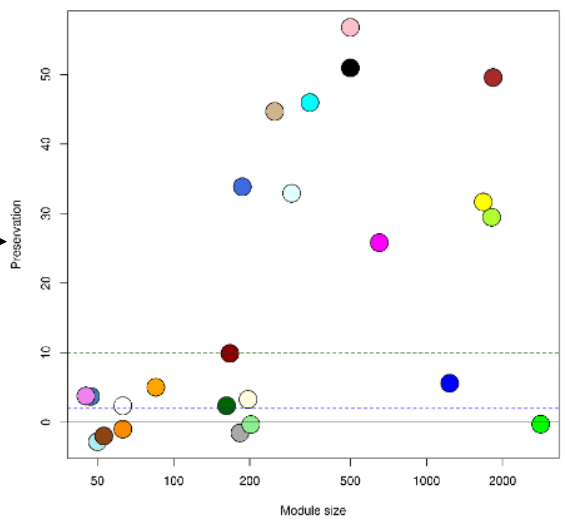

E

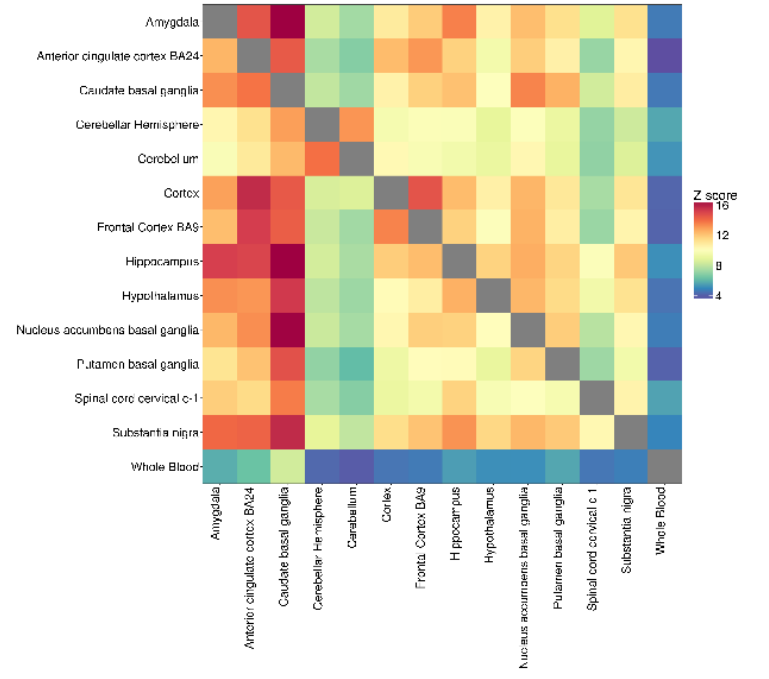

Supplement: S1 Fig — A. Build tissue-specific gene co-expression networks using Weighted Gene Co-expression Network Analysis. B. Test for the enrichment of GWAS signals using gene-based tests of association; C. Perform gene pathway analyses on GWAS enriched modules; D. Calculate the preservation of network module structure across tissue pairs; E. Summarize the preservation and stability of networks modules enriched with GWAS association signals. (PDF) [file pgen.1008245.s013.pdf]

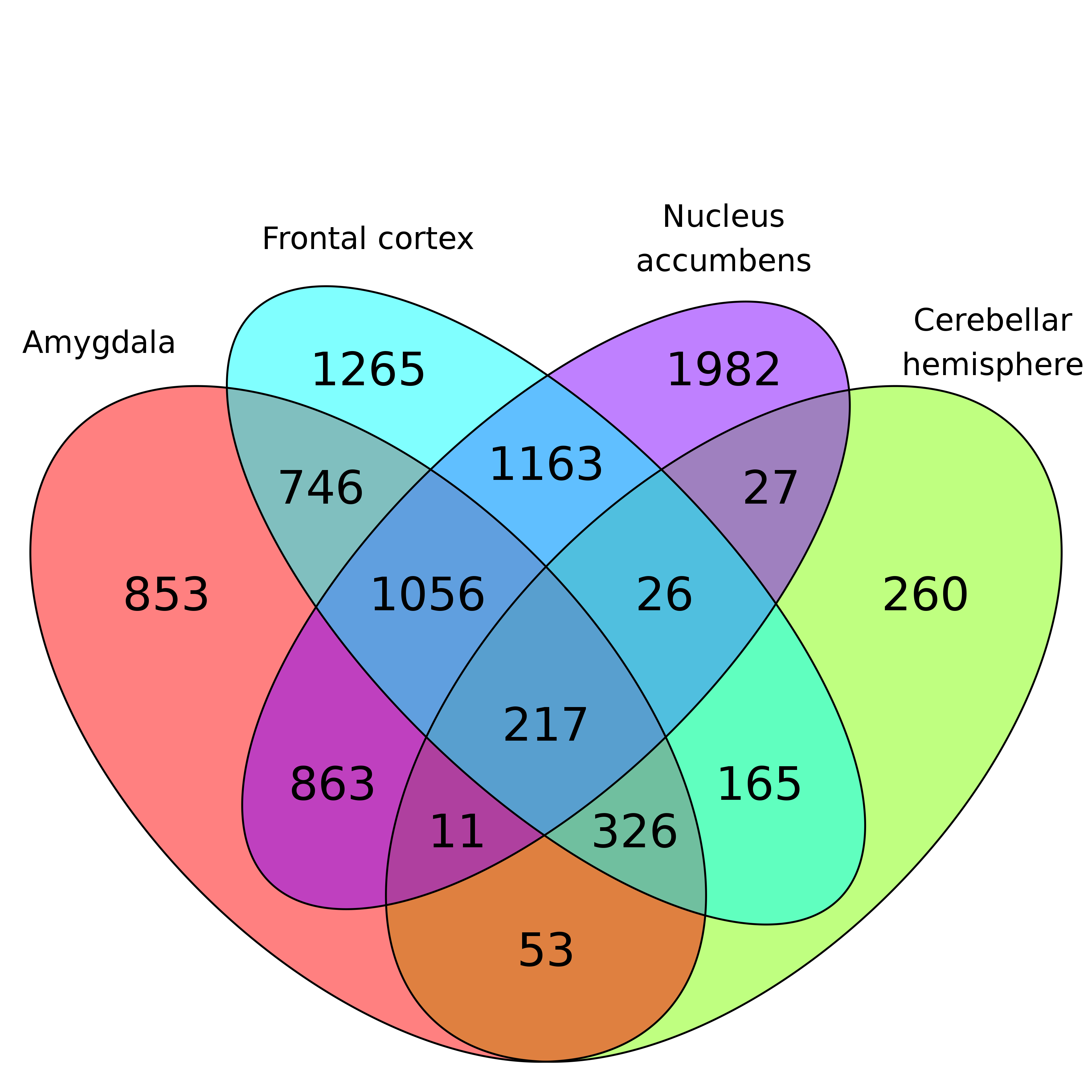

Supplement: S2 Fig — (TIFF) [file pgen.1008245.s014.tiff]
